# Optimization Design of MK-GGBS Based Geopolymer Repairing Mortar Based on Response Surface Methodology

**DOI:** 10.3390/ma16051889

**Published:** 2023-02-24

**Authors:** Zhiming Ma, Hancheng Dan, Jiawei Tan, Mengjin Li, Songlin Li

**Affiliations:** 1 School of Civil Engineering, Central South University, Changsha 410075, China; 2 Department of Civil Engineering, KU Leuven, 8200 Leuven, Belgium

**Keywords:** MK-GGBS-based geopolymer, repairing mortar, response surface method, coupling action

## Abstract

There are several influencing factors in the preparation of MK (metakaolin)-GGBS (ground granulated blast furnace slag)-based geopolymer repair mortars, including the MK-GGBS ratio, the alkalinity of the alkali activator solution, the modulus of the alkali activator solution, and the water-to-solid ratio. There are interactions between these factors, such as the different alkaline and modulus requirements of MK and GGBS, the interaction between the alkaline and modulus of the alkali activator solution, and the influence of water throughout the process. The effect of these interactions on the geopolymer repair mortar is not fully understood, making optimization of the MK-GGBS repair mortar ratio difficult. Therefore, in this paper, the response surface methodology (RSM) was used to optimize the preparation of the repair mortar, with GGBS content, SiO_2_/Na_2_O molar ratio, Na_2_O/binder ratio, and water/binder ratio as influencing factors and 1 d compressive strength, 1 d flexural strength, and 1 d bond strength as evaluation indices. Additionally, the repair mortar’s overall performance was assessed in terms of setting time, long-term compressive and bond strength, shrinkage, water absorption, and efflorescence. The results show that RSM was successful in establishing a relationship between the repair mortar’s properties and the factors. The recommended values of the GGBS content, Na_2_O/binder ratio, SiO_2_/Na_2_O molar ratio, and water/binder ratio are 60%, 10.1%, 1.19, and 0.41, respectively. The optimized mortar meets the standard’s requirements for set time, water absorption, shrinkage values, and mechanical strength, with minimal visual efflorescence. The back-scattered electron (BSE) images and energy dispersive spectroscopy (EDS) analysis show that the geopolymer and cement have good interfacial adhesion, and a denser interfacial transition zone exists in the optimized proportion.

## 1. Introduction

The deterioration of concrete structures has piqued the interest of civil engineers worldwide [1,2,3]. New repair materials are constantly being developed to strengthen and repair deteriorated concrete structures. Geopolymer is a new environmentally friendly cementing material that can set and harden quickly with high early strength, good long-term performance, durability, and a low carbon footprint [4]. Geopolymers are thought to be able to replace Portland cement as new building cementing materials. In recent years, because the elastic modulus and Poisson’s ratio of geopolymers are close to cement concrete [5,6] and it can bond well with cement concrete, people have gradually developed the application of geopolymer in concrete structural repair [7].

The original geopolymer repair materials were mostly based on MK, which possesses high late strength [8], good bonding capability [8,9], and good durability [10,11]. However, the necessity for high-temperature curing [12], poor early strength [8], poor flowability [13], and severe efflorescence substantially limit the use of MK-based polymers as repair materials [14]. Subsequent researchers found that the geopolymer could cure at room temperature, yielding a higher compressive strength by replacing MK with a specific amount of GGBS [15,16,17]. Hu et al. [17] found that a 20% GGBS replacement rate increased the 8 h strength by 43% and the 28-day strength by 7.6% compared with the MK-based geopolymer. This is because the C-(A)-S-H gel produced by GGBS can be combined with the N-A-S-H gel produced by MK, resulting in a dense structure [18,19]. Furthermore, bond strength, flowability, and weatherability were all improved when GGBS was used as a partial replacement for MK in geopolymer repair mortars [14,15,20]. The combination of MK and GGBS is clearly superior as a repair material.

The MK-GGBS-based geopolymer is affected by many factors, such as the ratio of MK and GGBS, the type and concentration of alkali activator solution [21], etc. Chen et al. [15] found that the geopolymer strength was greatest at 30% GGBS replacement rate. Tan et al. [14] found that a 10% GGBS replacement rate resulted in less strength loss after hydrochloric acid erosion, whereas a 30% GGBS replacement rate resulted in stronger freezing–thawing resistance and lower efflorescence risk. T. Pho-Ngernkham et al. [22] found that when NaOH and Na_2_SiO_3_ were used together as alkali activators, Na_2_SiO_3_ provides [SiO_4_]^4−^ to facilitate the geopolymerization reaction, and NaOH provides an alkaline environment to leach [SiO_4_]^4−^ and [AlO_4_]^5−^ for later reactions. G.F. Huseien et al. [16] discovered that if there was too much [SiO_4_]^4−^ in the solution, it would adsorb on the surface of the reactant particles and slow down the reaction. They believed that the combination of NaOH and Na_2_SiO_3_ is beneficial to alkali activators, but the proportions of NaOH and Na_2_SiO_3_ should be carefully controlled.

Meanwhile, there are coupling effects between these components as well. According to C.K. Yip et al. [23], at low alkali concentrations, the products of the MK and GGBS precursors coexisted, whereas the primary phase (i.e., N-A-S-H gel) generated by tiny calcium precipitation dispersed in the binder at a high alkali equivalent. Additionally, the concentration of alkali affects how easily Ca^2+^ dissolves. When the concentration of alkali is too high, Ca(OH)_2_ is formed, making the system unstable [24]. MK has a large flake surface area, consumes more free water early in the reaction, and releases less water in the late reaction, resulting in a denser structure than GGBS. However, its special flake structure also restricts its fluidity, resulting in a non-dense structure [16,25]. This indicates that the MK/GGBS ratio should take into account the effects of alkali concentration and water. Furthermore, as previously stated, NaOH and [SiO_4_]^4−^ in the alkali activator solution interact with each other, and water influences both concentrations. This demonstrates that each factor influences the others in the preparation of the geopolymer, and the coupling effects between all factors should be considered. However, most of the current studies only used a single factor or orthogonal test, which lacks the consideration of such a coupling effect.

RSM is a method to optimize experimental conditions. It can solve multi-factor and multi-level continuous response problems. The functional relationship between the influencing factors and the response values can be established by fitting the regression equation and drawing the response surface and contour line. Meanwhile, according to the response values of each factor level, the optimal predictive value is found [26]. RSM has previously been used to optimize cement production [27,28], and it is now being used to produce geopolymers [26,29]. However, research on the selection of repair materials is lacking including the short-term and long-term properties of mortars. In particular, extra alkali in the geopolymer framework tends to react with atmospheric carbon dioxide [30], resulting in efflorescence (the efflorescence process can be simplified as Equation (1)). Efflorescence may cause the geopolymer’s strength and stability to decline quickly. It is important to take into account the efflorescence resistance performance of the repair mortar.

(1) $$2NaOH+CO_{2}+H_{2}O\to Na_{2}CO_{3}+2H_{2}O$$

The purpose of this paper is to elucidate the variation regulation and mechanism of MK-GGBS-based geopolymer mortar under the coupling of various influencing factors, and to obtain the optimal mix ratio by RSM. In the proportioning experiments, the main influencing factors were GGBS content, Na_2_O/binder ratio, SiO_2_/Na_2_O molar ratio, and water/binder ratio, and the performance was evaluated using 1 d compressive strength, 1 d flexural strength, and 1 d bond strength. Setting time, shrinkage, water absorption, efflorescence, and long-term compressive and bond strength tests were performed to evaluate the performance of the optimized mortar. The BSE and EDS were used to characterize the adhesion of the geopolymer to the cement interface. The findings of this paper will aid in further optimizing the mix ratio of MK-GGBS-based geopolymer repair mortar as well as understanding its comprehensive performance as a repair material.

## 2. Materials and Methods

### 2.1. Raw Materials

**Figure 1:**
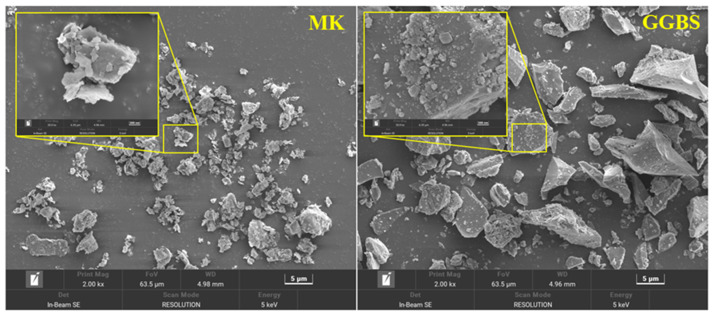
Morphology of the precursors.

**Figure 2:**
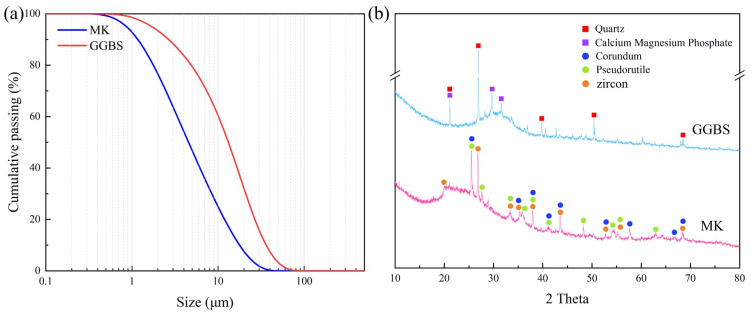
Precursor property: (**a**) size distribution of the precursors; (**b**) X-ray diffraction patterns of the precursors.

MK, GGBS, and ordinary Portland cement (OPC) used in this study are all commercially available materials. The chemical composition of the precursor was determined by X-ray fluorescence spectrometer (XRF), as shown in Table 1. MK has the same SiO_2_ content as GGBS, but MK has more Al_2_O_3_ and GGBS has more CaO. The microstructure of MK and GGBS is shown in Figure 1. MK is a flake and GGBS is an angular particle. Figure 2a and Table 2 show the particle size of the precursors, with the mean particle size (D50) of MK and GGBS being 4.5 μm and 13.17 μm, respectively. Figure 2b shows the XRD pattern of the precursor. The main crystalline phases of MK are corundum, pseudorutile, and zircon, while the main crystalline phases of GGBS are quartz and calcium magnesium phosphate. International Standards Organization (ISO) sand is used as fine aggregate, and its physical properties are shown in Table 3. Sodium hydroxide (alkali flakes, purity greater than 99%), sodium silicate (27.3 wt.% SiO_2_, 8.54 wt.% Na_2_O, and 64.16 wt.% H_2_O), and tap water were used to prepare the alkali activator.

### 2.2. Experimental Design by RSM

The Box Behnken design (BBD) and Central Composite design (CCD) methods are commonly used in RSM. BBD can reduce the number of experiments with the same factors and levels, and CCD can better fit the surface by taking extreme points; however, taking extreme values leads to poor test results because the factor levels are outside the normal range, so the BBD method was used to ensure test accuracy. GGBS content (factor A), Na_2_O/binder ratio (factor B), SiO_2_/Na_2_O molar ratio (factor C), and water/binder ratio (factor D) were selected as influencing factors, with three levels for each factor. The value was determined by the preliminary experiment, as shown in Table 4. In order to utilize the advantages of fast setting and high early strength of the MK-GGBS-based geopolymer in the field of rapid repair, the 1 d compressive strength ($y_{1}$), 1 d flexural strength ($y_{2}$), and 1 d bond strength ($y_{3}$) were selected as the evaluation indices in combination with the requirements of the Chinese standard for repair mortars (JC/T 2381-2016) and the Chinese technical specifications for pavements (JTJ0731-2001 and JTG D40-2011). The specific test scheme is shown in Table 5.

### 2.3. Sample Preparation

Geopolymer mortar was prepared according to Table 5. The alkali activator solution was first prepared by mixing sodium hydroxide with water and stirring well to obtain a sodium hydroxide solution, then the water glass was added to the sodium hydroxide solution and stirred well and cooled for 24 h in a laboratory environment. To make the mortar, MK, GGBS and sand were mixed together for 2 min at low speed to create a homogeneous mixture of solids. Then, alkali activator solution was added and mixed at low speed for 2 min, then at high speed for 2 min. The fresh mortar is injected into the mold in two stages, with each vibration lasting 60 strokes. After vibration, it is be covered with cling film and cured in the laboratory environment (temperature: 25 °C, humidity: 60%) until testing.

### 2.4. Test Methods

The setting time test was based on the Chinese national standard GB/T 50080 test setting time with a penetration resistance meter. The test of compressive and flexural strength was based on the Chinese national standard GB/T 17671-2021. For the flexural strength test, mortar samples of 40 mm × 40 mm × 160 mm are used. A pressure testing machine (TSY-2000 constant loading pressure testing machine) was used for loading, and the loading rate was 50 N/s. Three samples were prepared for each group, and the test results were averaged. The compressive strength of the broken prisms is tested. The compressive surface is the two sides of the test body when it is formed, and the area is 40 mm by 40 mm. A pressure testing machine (TSY-2000 constant loading pressure testing machine) was used for loading, with a loading rate of 2.4 KN/s. Six samples were prepared for each group, and the test results were averaged.

**Figure 3:**
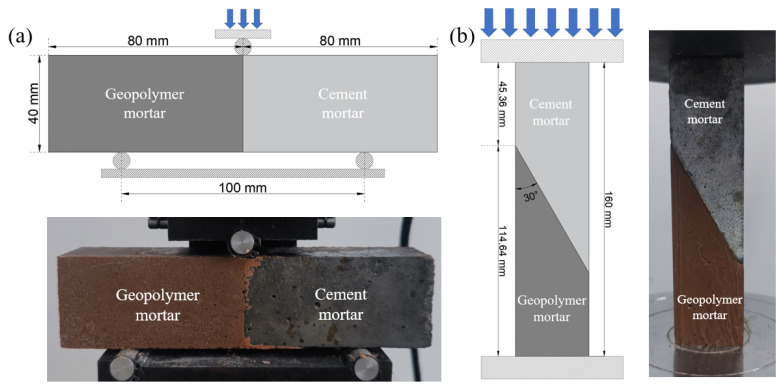
Schematic diagrams: (**a**) flexural bending strength test; (**b**) slant shear test.

The bond strength test is shown in Figure 3. Two methods were used to test the bond strength. The slant shear test was used to evaluate the strength value [31], and the flexural bending strength test was used to evaluate the adhesion effect and failure mode of the interface. The cement mortar was first created as a substrate by filling one side of the 40 mm × 40 mm × 160 mm mold with extruded polystyrene foam and the cement mortar (sand:cement:water = 3:1:0.5) was poured on the other side, and then demolded and conditioned in a laboratory environment for 28 days. The cement substrate was then placed on one side of the 40 mm × 40 mm × 160 mm mold, and the bonded surface had been sandpapered smooth in advance to eliminate the effect of roughness [8]. In the other side a geopolymer mortar was poured, and then it was demolded and cured in a laboratory environment until tested. After curing in the laboratory environment, a pressure testing machine (TSY-2000 constant loading pressure testing machine) was used for loading according to the loading method shown in Figure 3, and the loading rate was 50 N/s. Equations (2) and (3) were used to calculate the slant shear strength and flexural bending strength. A set of three specimens were averaged to determine the bond strength value.

(2) $$f_{s}=\frac{F\surd 3}{6400}$$

(3) $$f_{f}=\frac{75F}{32}$$

where $f_{s}$ is the slant shear strength (MPa), $f_{f}$ is the flexural bending strength (MPa), and *F* is the loading force when the sample fails (N).

The water absorption test was based on the Chinese standard JG/T 2381-2016. A 40 mm × 40 mm × 80 mm geopolymer mortar specimen was prepared, of which a 40 mm × 40 mm surface was the test surface. After curing for 28 d in the laboratory environment, the specimen was put into a 70 °C blast drying oven and dried to a constant weight. After cooling to room temperature, five surfaces except the test surface were coated with sealing materials, and the mass $\;m_{a}$ of the specimen was weighed. The specimen was vertically placed on a 25 g/L~30 g/L saturated polyurethane sponge filled with water, face down. At 6 h and 72 h, the surface of the specimen was wiped dry to measure the mass ($m_{b}$). The water absorption of the mortar was calculated according to Equation (4):

(4) $$W_{ab}=\frac{m_{b}-m_{a}}{1.6}$$

where $W_{ab}$ is the amount of water absorbed in 6 h or 72 h (kg/m^2^), $m_{a}$ is the mass of the specimen before immersion (g), and $m_{b}$ is the mass of the specimen after immersion (g).

The shrinkage test of the mortar was based on the Chinese standard JC/T 603. When the specimen’s compressive strength reached (10 ± 2) MPa, the initial length $L_{0}$ of the test piece was measured within 30 min of its release. The length of the specimen was measured at 1 d–7 d, 14 d, and 28 d, denoted as $L_{t}$. The shrinkage rate of the mortar was calculated according to Equation (5):

(5) $$S_{t}=\frac{\left(L_{0}-L_{t}\right)\times 100}{250}$$

where $S_{t}$ is the shrinkage rate at time *t* (%), *t* = 1–7 d, 14 d, and 28 d, $L_{0}$ is the initial measurement reading (mm), $L_{t}$ is the measured reading at time *t* (mm), and 250 is the effective length of the specimen (mm).

**Figure 4:**
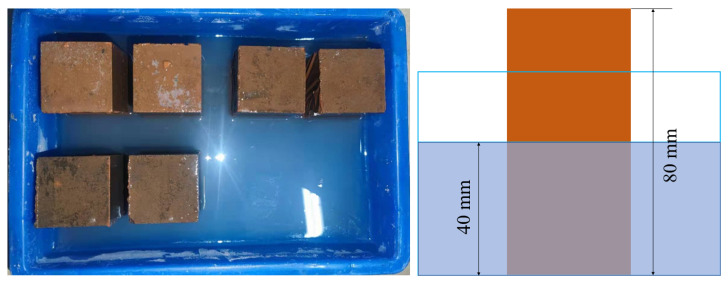
Efflorescence test.

The efflorescence of materials was assessed by visual observation [32]. The mortar test of 40 × 40 × 80 mm was placed vertically in water with a depth of 40 mm along the length direction of 80 mm after curing for 28 d, as shown in Figure 4. After curing for 28 d in the laboratory environment, the efflorescence degree of the samples was observed visually.

**Figure 5:**
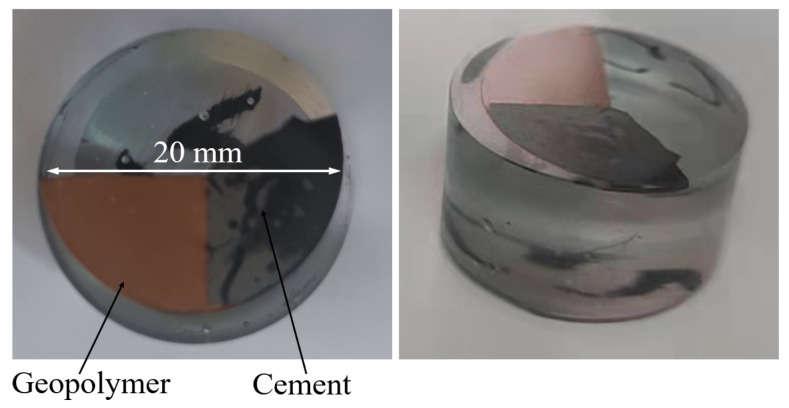
Sample preparation for the BSE test.

BSE and EDS observed the interface between the geopolymer-repairing sample and matrix, as shown in Figure 5. First, the cement sample (cement:water = 1:0.5) was prepared, cured for 28 d in the laboratory environment, and then poured into the geopolymer slurry. After curing for 24 h, isopropyl alcohol was used to terminate the hydration of the obtained samples [14]. The sample was dried and then inlaid with epoxy resin under vacuum. An automatic polisher was used to sand and polish the sample until a smooth surface was obtained.

## 3. Results and Discussion

### 3.1. RSM Results and Analysis

According to the 29 combinations in Table 5, 1 d compressive strength, 1 d flexural strength, and 1 d bond strength of geopolymer mortar were tested, and the test results are shown in Table 6.

Design-Expert software was used to analyze the applicability of the mathematical model obtained from the test data, and the analysis of variance (ANOVA) results of the model were obtained, as shown in Table 7. In Table 7, the interaction of factors A and B are represented in the table, and the symbols in the table represent factors and the interaction between factors.

Table 7 shows that the *p* value for the “model” terms for 1 d compressive strength, 1 d flexural strength, and 1 d bond strength are less than 0.0001, while the *p* value for “Lack of Fit” < 0.05. *p* values are commonly used to validate the significance of the regression coefficient, and models with *p* < 0.05 are generally considered significant, while models with *p* > 0.05 are considered insignificant. This indicates that the three regression models $y_{1}$, $y_{2}$, and $y_{3}$ are significant, while their misfit terms are insignificant. In addition, the R^2^ of the three models are 0.9849, 0.9844, and 0.9758, respectively, and the adjusted R^2^ are 0.9697, 0.9688, and 0.9515, respectively, which are all greater than 0.95, indicating that these models can effectively explain the changes in response values and are suitable for predictive analysis, and the analysis was conducted at the 5% level of significance. Based on the results of the experiment, the prediction functions of $y_{1}$, $y_{2}$, and $y_{3}$ were obtained by ANOVA as shown in Equations (6)–(8):

(6) $$y_{1}=40.17+4.76A+3.37B-0.74C-4.66D+0.1AB-2.20AC-2.64AD+1.3BC-0.35BD-3.98CD+1.09A^{2}-9.42B^{2}-7.93C^{2}-5.86D^{2}$$

(7) $$y_{2}=7.21+0.75A+0.56B-0.19C-0.94D+0.28AB-0.28AC+0.64AD+0.052BC-0.095BD-0.95CD-0.19A^{2}-1.37B^{2}-1.16C^{2}-0.84D^{2}$$

(8) $$y_{3}=4.44+0.62A+0.39B-0.35C-1.21D-0.29AB-0.33AC+0.13AD+0.027BC-0.20BD-0.39CD-0.28A^{2}-0.77B^{2}-0.82C^{2}-0.41D^{2}$$

### 3.2. Effect of the Variables on the Mechanical Strength of the Repairing Mortar

#### 3.2.1. Compressive Strength of 1 d

According to the “*p*-value” in Table 7, it is clear that for 1 d compressive strength, factors A, B, and D have a highly significant effect (*p* < 0.0001), and factor C has a non-significant effect (*p* > 0.05). Meanwhile, there is a significant interaction between factors A × C, factors A × D, and factors C × D. Based on the magnitude of the “F-value”, it can be seen that the significant effects of each factor on 1 d compressive strength is as follows: A > D > B > C×D > A×D > A×C.

**Figure 6:**
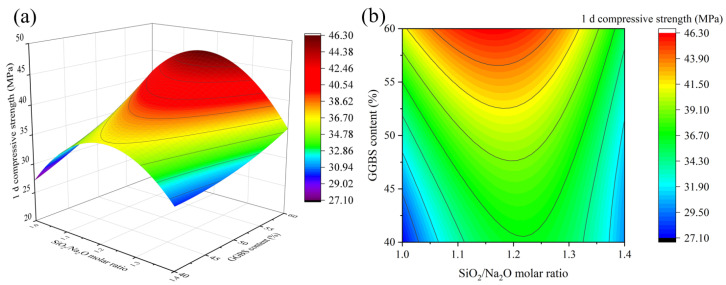
The interaction effect of SiO_2_/Na_2_O molar ratio and GGBS content on 1 d compressive strength: (**a**) 3D response surface; (**b**) contour diagram.

**Figure 7:**
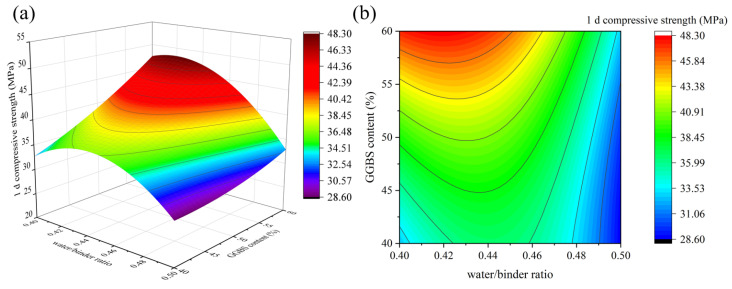
The interaction effect of water/binder ratio and GGBS content on 1 d compressive strength: (**a**) 3D response surface; (**b**) contour diagram.

**Figure 8:**
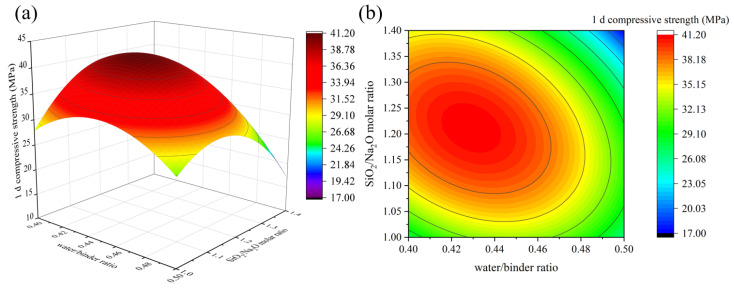
The interaction effect of water/binder ratio and SiO_2_/Na_2_O molar ratio on 1d compressive strength: (**a**) 3D response surface; (**b**) contour diagram.

Figure 6, Figure 7 and Figure 8 depict the influence of the interaction between factors on 1 d compressive strength value. Factors other than horizontal and vertical coordinates are taken at the intermediate level. The results in Figure 6 and Figure 7 show that when the SiO_2_/Na_2_O molar ratio and the water/binder ratio are fixed, the 1 d compressive strength increases with the increase in the GGBS content. The reason is that Ca^2+^ formed after GGBS dissolution can quickly generate C-S-H gel and calcium (alumino) silicate hydrate (C-(A)-S-H) gel with [SiO_4_]^4−^ in sodium silicate and [AlO_4_]^5−^ in solution [33]. Figure 8 shows that when the GGBS content is fixed, with the increase in the SiO_2_/Na_2_O molar ratio, the 1 d compressive strength of geopolymer first increases and then decreases, reaching the maximum value between 1.15 and 1.25, which is close to the results of many studies [4,7]. In fact, the dissolution of silicate monomer in the precursor is more difficult than that of aluminate monomer. Therefore, the introduction of sodium silicate can provide soluble silicate to accelerate the geopolymer reaction [22]. However, when the SiO_2_/Na_2_O molar ratio is too high, the alkali concentration will be reduced accordingly, and the generated silicate monomer will also be adsorbed on the surface of solid particles, hindering the reaction. Figure 7 shows that when GGBS content is fixed, the strength of geopolymer increases slightly and then decreases, reaching the maximum value between 0.42 and 0.44, which is related to the low flowability of geopolymer when the ratio of MK is high. Appropriately increasing the water/binder ratio can improve its fluidity, and expand the spacing between particles to promote dissolution and make the structure denser. Further increase in water will result in increased porosity and decreased structure strength. Figure 8 shows that with the increase in the SiO_2_/Na_2_O molar ratio and the water/binder ratio, the 1 d compressive strength first increases and then decreases. When the SiO_2_/Na_2_O molar ratio is 1.15~1.25 and the water/binder ratio is 0.4~0.44, the maximum values can be obtained. When the SiO_2_/Na_2_O molar ratio is 1.4 and the water/binder ratio is 0.5, the compressive strength is only 17 MPa, which is because the alkalinity of the solution is low and the precursors cannot be fully excited [16].

#### 3.2.2. Flexural Strength of 1 d

According to the “*p*-value” value in Table 7, factor C has a significant effect on 1 d flexural strength when compared with 1 d compressive strength (*p* = 0.0133), and there is also an interaction between factor A × B. Based on the magnitude of the “F-value”, it can be seen that the significance of each factor on 1 d flexural strength is as follows: D > A > B > C×D > A×D > C > A×B > A×C.

**Figure 9:**
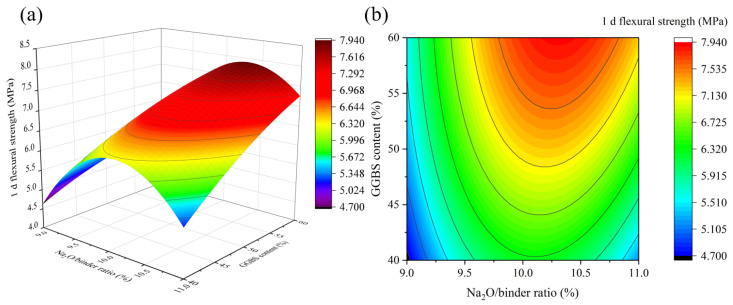
The interaction effect of Na_2_O/binder ratio and GGBS content on 1 d flexural strength: (**a**) 3D response surface; (**b**) contour diagram.

**Figure 10:**
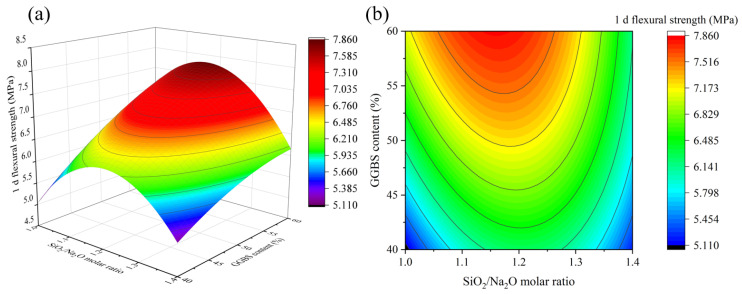
The interaction effect of SiO_2_/Na_2_O molar ratio and GGBS content on 1 d flexural strength: (**a**) 3D response surface; (**b**) contour diagram.

**Figure 11:**
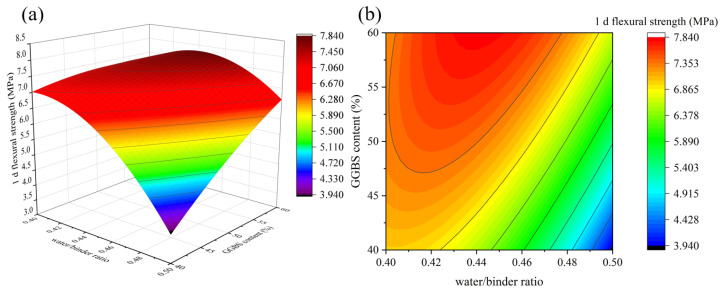
The interaction effect of water/binder ratio and GGBS content on 1 d flexural strength: (**a**) 3D response surface; (**b**) contour diagram.

**Figure 12:**
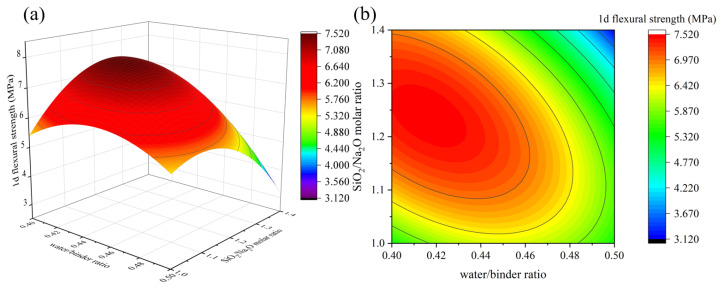
The interaction effect of water/binder ratio and SiO_2_/Na_2_O molar ratio on 1 d flexural strength: (**a**) 3D response surface; (**b**) contour diagram.

Figure 9, Figure 10, Figure 11 and Figure 12 depict the influence of the interaction between factors on 1 d flexural strength. Figure 11 shows that with the increase in the Na_2_O/binder ratio, 1 d flexural strength first increases and then decreases, and the maximum value is reached when the Na_2_O/binder ratio is 10~10.5%, which shows a consistent rule under different GGBS contents. An appropriate Na_2_O/binder ratio can provide a suitable alkaline environment for the dissolution of solid precursors. If the Na_2_O/binder ratio is too low, the efficiency of the solid dissolution of the silicaluminate monomer is low; if the ratio is too high, it will cause the dissolution of the silicaluminate oligomer and the reduction in free water, thus reducing the reaction rate [34]. The rule in Figure 10, Figure 11 and Figure 12 are consistent with the results of 1 d compressive strength. Especially in Figure 14, when the GGBS content is 60%, the decline in the flexural strength with the water/binders ratio is significantly slower than that when the GGBS content is 40%. Obviously, more GGBS incorporation effectively improves the impact of the increase in the water/binder ratio on 1 d flexural strength.

#### 3.2.3. Bond Strength of 1 d

According to the “*p*-value” in Table 7, the 1 d bond strength and 1 d flexural strength are consistent in terms of the significance of the single factors. In the interaction between factors, there are significant interactions between factors A×B, factors A×C, and factors C×D. The significance of each factor of 1 d bond strength is as follows: D > A > B > C > C×D > A×C > A×B.

**Figure 13:**
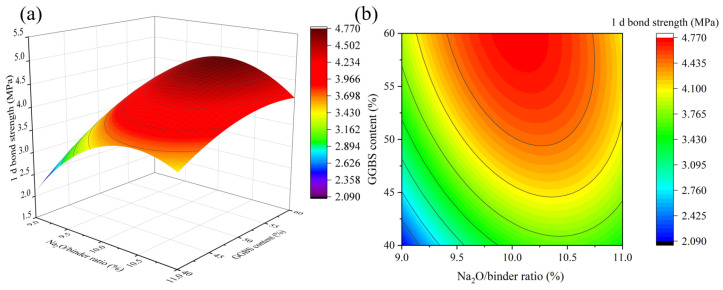
The interaction effect of Na_2_O/binder ratio and GGBS content on 1 d bond strength: (**a**) 3D response surface; (**b**) contour diagram.

**Figure 14:**
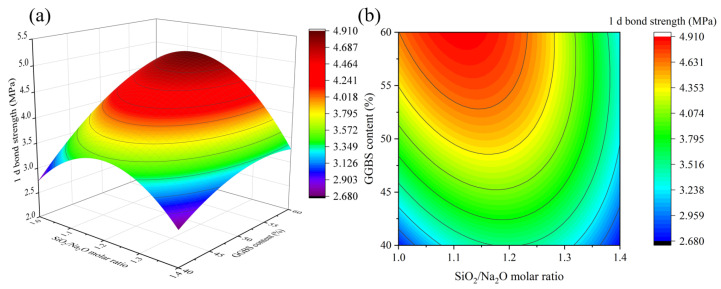
The interaction effect of SiO_2_/Na_2_O molar ratio and GGBS content on 1 d bond strength: (**a**) 3D response surface; (**b**) contour diagram.

**Figure 15:**
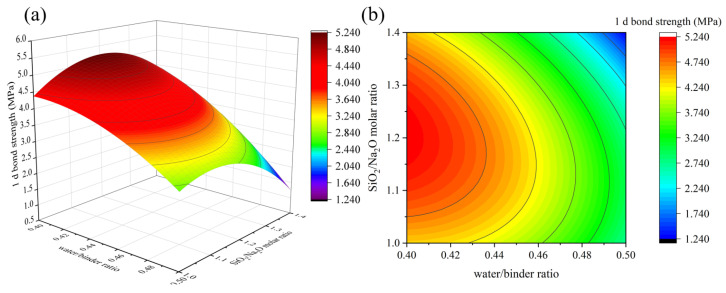
The interaction effect of water/binder ratio and SiO_2_/Na_2_O molar ratio on 1 d bond strength: (**a**) 3D response surface; (**b**) contour diagram.

Figure 13, Figure 14 and Figure 15 depict the influence of the interaction of factors on 1 d bond strength. Figure 13 shows that as the Na_2_O/binder ratio increases, the 1 d bond strength increases first and then decreases, reaching its maximum value when the Na_2_O/binder ratio is between 10% and 10.5%. Different from 1 d flexural strength, when 1 d bond strength exceeds the peak value, the bond strength declines more slowly as the Na_2_O/binder ratio continues to increase, which may be due to the reaction of part of the alkali solution with the cement matrix [35]. This indicates that a high Na_2_O/binder ratio is beneficial for bond strength. The results of Figure 14 and Figure 15 are consistent with those of compressive and flexural resistance. In particular, it was found that, compared with the 1 d compressive strength and 1 d flexural strength, the 1 d bond strength decreased more slowly in the SiO_2_/Na_2_O molar ratio from 1.2 to 1.0, which is consistent with Figure 15. Lower SiO_2_/Na_2_O molar ratios corresponded to higher Na_2_O content. This further illustrates the benefits of higher alkalinity for bonding.

### 3.3. RSM Solving and Verification

**Figure 16:**
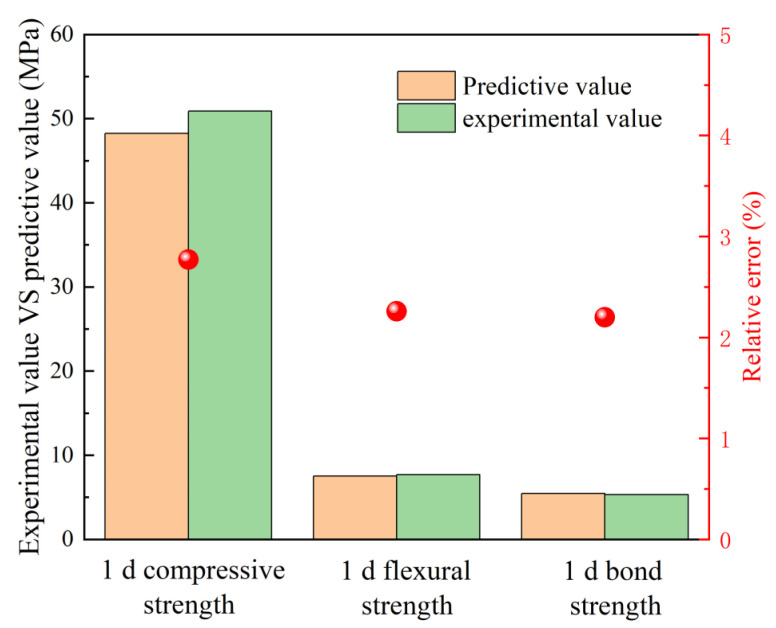
Verification of RSM in the optimal condition.

In the Design-Expert software, the target of 1 d compressive strength, 1 d flexural strength, and 1 d bond strength was set as the maximum, and the levels of each factor were obtained as follows: A = 60%, B = 10.2%, C = 1.19, and D = 0.41, and the ratio was recorded as Mix RSM. In order to verify the prediction model of RSM, tests were carried out again for Mix RSM, three times for each group, and the average value was taken. The test results are shown in Figure 16. The results show that the relative error between the predicted values of the model and the experimental results is less than 3%. The results predicted by the model can reflect the real-life test situation.

### 3.4. Performance Test of Geopolymer Repairing Mortar

The results of RSM show that GGBS content is the main factor affecting the properties of the geopolymer repair mortar. This is related to the change in gel composition of the geopolymer when MK and GGBS are mixed in a certain proportion [36], and correspondingly, its performance as a repair material will also change. In order to explore the other properties of Mix RSM repair mortar and the influence of GGBS content, for subsequent studies, the Na_2_O/binder ratio, SiO_2_/Na_2_O molar ratio, and water/binder ratio are recommended values, and the GGBS content should be set at 0, 60%, and 100% (labeled Mix MK, Mix RSM, and Mix GGBS, respectively).

#### 3.4.1. Setting Time

**Figure 17:**
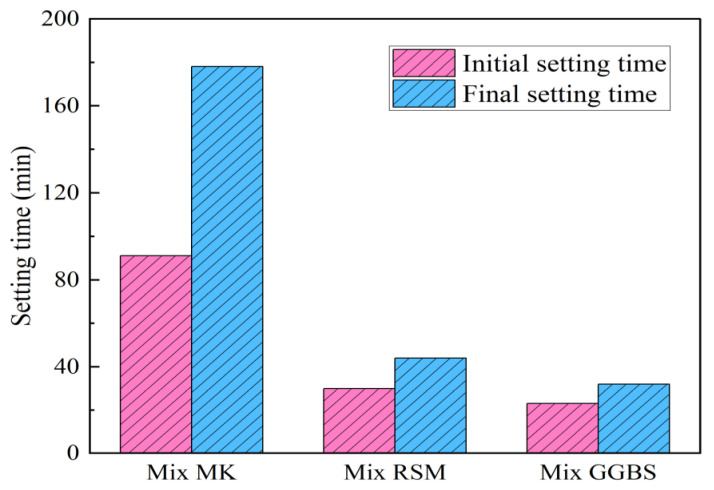
Setting time of geopolymer.

Figure 17 records the test results of the setting time. Compared with Mix MK, the initial setting time of Mix RSM and Mix GGBS is reduced by 67% and 75%, and the final setting time is reduced by 75% and 82%, respectively. Obviously, the incorporation of GGBS is beneficial for rapid coagulation. This is due to the higher bond energies of the Si-O and Al-O bonds, and it takes longer to break down. Conversely, the bond energy of Ca-O is lower and it can react with [SiO_4_]^4−^ in the alkali activator solution more quickly to form C-S-H gel and encourage coagulation [33,35,37]. The setting time of Mix RSM and Mix GGBS is similar, indicating that GGBS with a 60% replacement rate can achieve the same setting time as the GGBS-based geopolymer mortar. Meanwhile, Mix RSM and Mix GGBS meet the requirements of JC/T 2381-2016: initial setting time ≤ 30 min; final setting time ≤ 50 min.

#### 3.4.2. Mechanical Property

**Figure 18:**
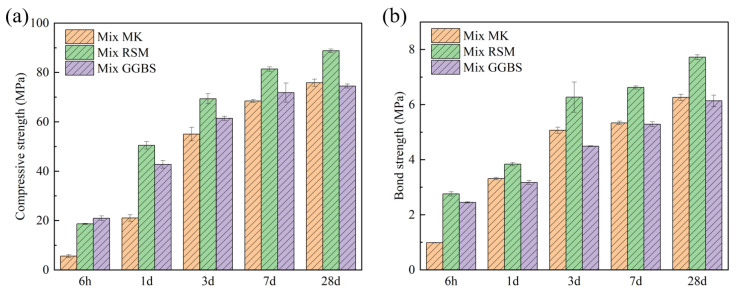
Mechanical properties test results: (**a**) compressive strength; (**b**) bond strength.

**Figure 19:**
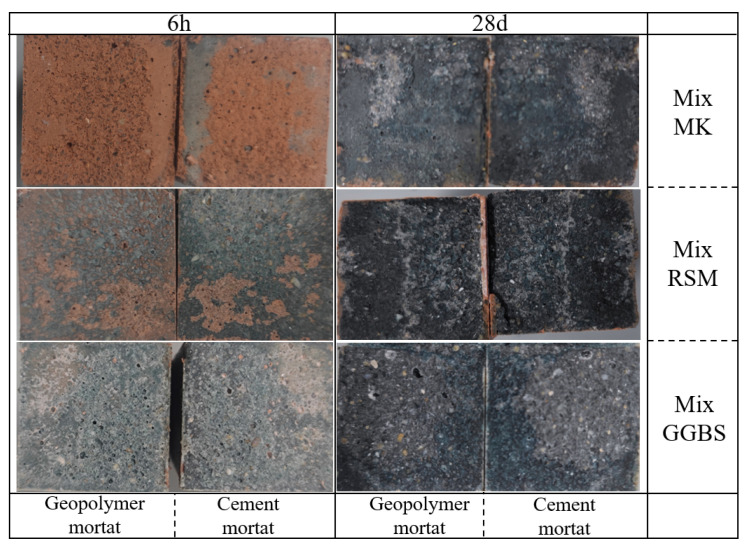
Fracture surface diagram of flexural bending strength specimen.

At 6 h, Mix MK has the lowest strength (5.58 MPa), while Mix GGBS has the highest strength (20.94 MPa). The high early strength of Mix GGBS is related to the mass generation of C-S-H gel and C-(A)-S-H gel [14,38], but due to the gel’s rapid generation, the gel will wrap around the reactant particles. As a result, the subsequent strength development was stymied, and the strength of Mix GGBS almost did not develop from 7 d to 28 d, while the strength of Mix MK exceeded the strength of Mix GGBS in this case. The intensity of the Mix RSM was high (18.65 MPa at 6 h) in the early stage of the reaction due to the presence of more GGBS, and continued to increase in the late stage due to the presence of MK. Mix RSM has the highest strength from 1 d to 28 d because the gels produced by MK and GGBS can be combined to make the structure more dense. The bond strength of the geopolymer mortar test results is depicted in Figure 18b. The bond strength followed a similar pattern to the compressive strength, with the exception that at 1 d, the bond strength of Mix MK was already higher than that of Mix GGBS. Table 8 records the compressive and bond strengths specified in JC/T 2381-2016. Therefore, the compressive and bond strengths of Mix RSM and Mix GGBS meet the requirements. Figure 19 depicts the bond strength specimens breaking. The specimens all break on the geopolymer side at 6 h, but Mix RSM has less geopolymer adhering to the cement side than Mix MK and Mix GGBS, which is consistent with the test results of bond strength. At 28 d, the specimens all break on the cement matrix side, indicating that the geopolymer’s adhesion strength to the cement matrix exceeds the cement matrix’s strength. Table 9 records the bond strength values of the repair mortar tested by some scholars [15,31,38,39,40,41,42], and the method adopted is consistent with this study. Geopolymer repair mortar has a higher bonding strength than some conventional and modified repair materials. Although magnesium phosphate cement (MPC) repair mortar has a strength of 4.1 MPa in 1 d, its preparation via an acid–base reaction is costly, and its compressive strength in contact with water is greatly reduced [43], severely limiting its application. In addition, compared with the bond strength of other geopolymers listed in Table 9, Mix RSM has higher bond strengths of 1 d and 28 d. Although different raw materials and preparation processes may lead to bias in the results, it also indicates that the RSM method can optimize the performance of geopolymer repair mortar to a certain extent.

#### 3.4.3. Shrinkage

**Figure 20:**
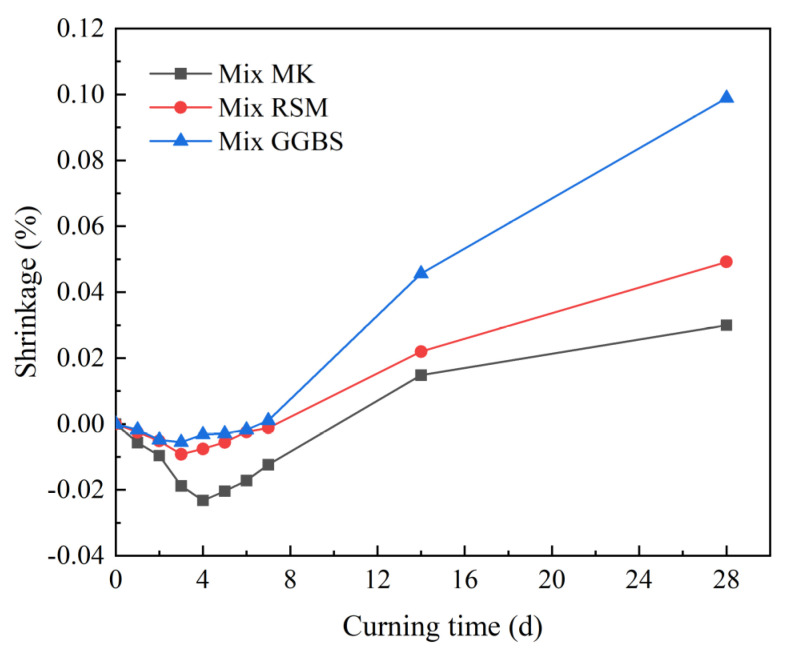
Shrinkage of geopolymer mortars.

The results of the shrinkage test are shown in Figure 20. In the first few days, the specimen would first expand, at which time the expansion of Mix MK was larger and the expansion value of Mix RSM was close to that of Mix GGBS. This is due to the geopolymer’s chemical swelling. Swelling in MK-based geopolymers may be caused by the continuous movement of water from the macropores into the mesopores, as well as salt precipitation and the formation of the crystalline zeolite phase, both of which can cause swelling [44,45,46]. Although shrinkage is the dominant phenomenon in GGBS-based geopolymers, there is also volume expansion due to crystalline phase swelling [47]. The swelling occurs early in the reaction, followed by shrinkage. At 28 d, the shrinkage value of Mix MK was the lowest (0.03%), and the shrinkage value of Mix GGBS was the highest (0.1%). The shrinkage value of Mix RSM was 0.05% in the middle, and all the shrinkage values met the 28 d shrinkage value ≤ 0.1% specified by JC/T 2381-2016.

#### 3.4.4. Water Absorption and Efflorescence

**Figure 21:**
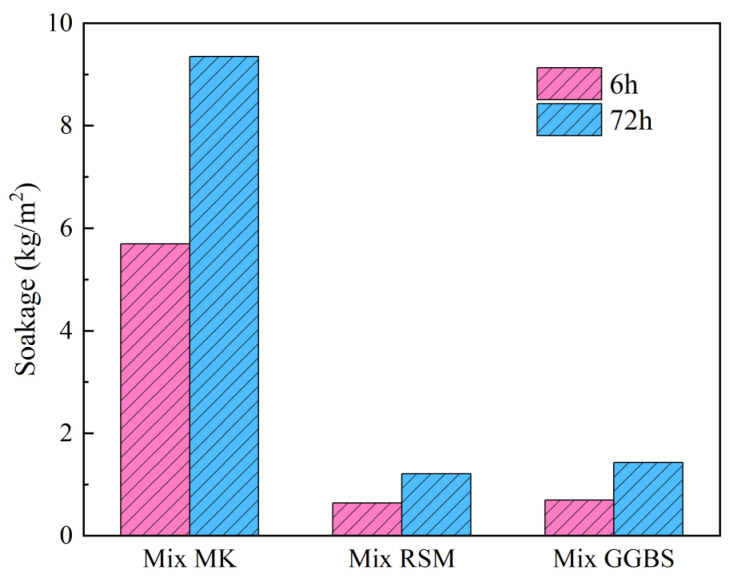
Soakage of geopolymer mortars.

**Figure 22:**
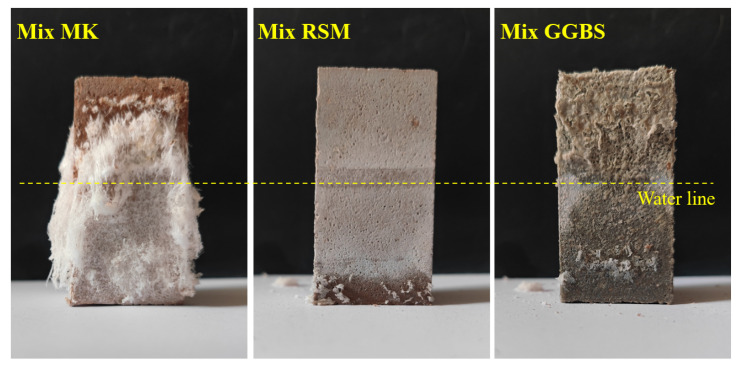
The visual efflorescence extent of samples.

Water absorption reflects the water resistance of the geopolymer mortar and is a reference for the repair of structures exposed to humid environments. Especially, because geopolymer is prone to efflorescence in humid environments, there is a certain relationship between water absorption and efflorescence resistance. Figure 21 shows the geopolymer’s water absorption over the course of 6 h and 72 h. According to the findings, Mix MK has a very high water absorption of 5.69 kg/m^2^ and 9.35 kg/m^2^ at 6 h and 72 h, which is much higher than the 1.2 kg/m^2^ and 2.0 kg/m^2^ recommended by JC/T 2381-2016. In contrast, water absorption is reduced in Mix RSM and Mix GGBS due to the denser structural makeup; for instance, the values of water absorption are respectively 0.64 kg/m^2^ and 0.69 kg/m^2^ at 6 h, while they are respectively 1.21 kg/m^2^ and 1.43 kg/m^2^ at 72 h. It should be pointed out that the values of water absorption of both Mix RSM and Mix GGBS meet the requirements of the standard. Figure 22 shows the result of the visual efflorescence of geopolymer mortar. Mix MK exhibits the most severe visual weathering and a significant amount of crystallization, followed by Mix GGBS. In contrast, Mix RSM only exhibits a white layer of lye bleed on the surface without any discernible weathering crystallization. It is in line with the findings regarding water absorption and indicates that the geopolymer’s water and weathering resistance is further improved when the ratio of MK to GGBS is suitable.

#### 3.4.5. BSE and EDS

**Figure 23:**
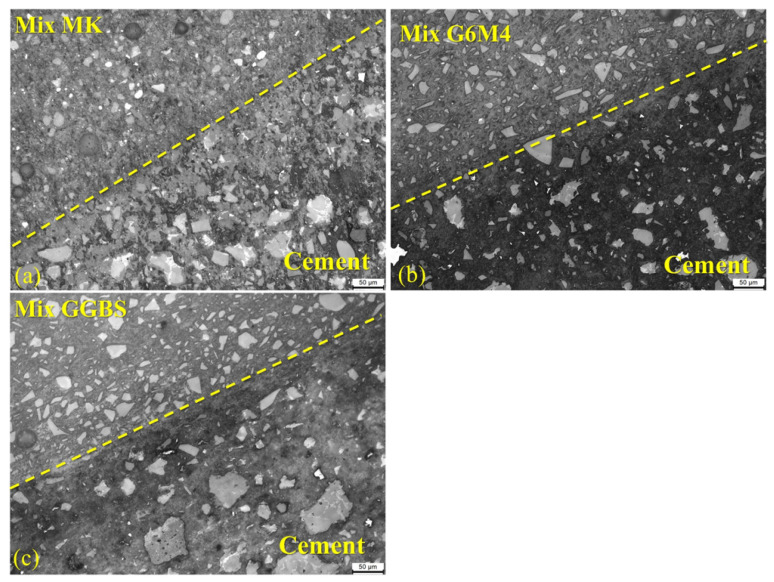
BSE diagram of bonding interface (24 h): (**a**) Mix MK; (**b**) Mix RSM; (**c**) Mix GGBS.

**Figure 24:**
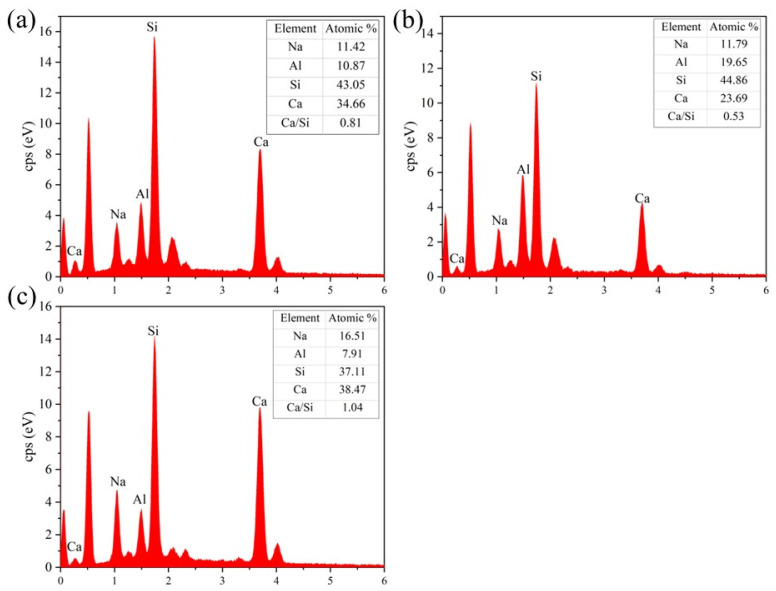
EDS diagram of bonding interface (24 h): (**a**) Mix MK; (**b**) Mix RSM; (**c**) Mix GGBS.

Figure 23 depicts the results of the BSE testing. The Mix RSM and Mix GGBS gels were denser and contained more unreacted particles than the Mix MK gels. This is related to the fact that the C-(A)-S-H gel is adsorbed on the surface of the reactant particles while filling the structure, preventing further development of the reaction [16,48]. Mix RSM has fewer unreacted large particles and more gel than Mix GGBS, which is the reason for the higher strength of Mix RSM. In addition, it can be seen that the interface between all groups and the cement is well bonded. This further illustrates the advantages of geopolymers as repair materials. The chemical elements of the interface are shown in Figure 24. The Ca/Si of Mix MK, Mix GGBS, and Mix RSM were 0.81, 1.04, and 0.53, respectively. Because the alkali activator can react with Ca(OH)_2_ in cement to produce low-Ca/Si C-S-H gels [35,49,50]. The Ca/Si of Mix RSM is the lowest, which means that there is more C-S-H gel and less Ca(OH)_2_ on the interface of Mix RSM, which makes the transition zone of the interface more dense. This is also the reason for the higher bond strength of Mix RSM.

## 4. Conclusions

In order to optimize the proportion of MK-GGBS-based geopolymer repair mortar, the RSM method was used to investigate the effects of GGBS content, Na_2_O/binder ratio, SiO_2_/Na_2_O molar ratio, and water/binder ratio, and their interactions on 1 d compressive strength, 1 d flexural strength, and 1 d bond strength. Setting time, long-term mechanical properties, shrinkage, water absorption, and efflorescence potential of the optimized geopolymer repair were discussed. The main conclusions are as follows:

- Based on RSM, the prediction models of 1 d compressive strength, 1 d flexural strength, and 1 d bond strength of MK-GGBS-based geopolymer mortar were established at the significant level of 5%. ANOVA analysis results showed that GGBS content, water/binder ratio, and Na_2_O/binder ratio had significant effects on each response, and SiO_2_/Na_2_O molar ratio had significant effects on 1 d flexural strength and 1 d bond strength; the effect on 1 d compressive strength was not significant.
- The RSM analysis results showed that the 1 d compressive strength, 1 d flexural strength, and 1 d bond strength were all proportional to the GGBS content, increasing and then decreasing with the increase in Na_2_O/binder ratio, SiO_2_/Na_2_O molar ratio, and water/binder ratio, with maximum values at 10%~10.5%, 1.15~1.25, and 0.42~0.44, respectively. When the SiO_2_/Na_2_O molar ratio was 1.4 and the water/binder ratio was 0.5, the alkalinity of the solution was low, the precursor could not be fully excited, and the 1 d compressive strength was only 17 MPa. When the water/binder ratio increased from the optimal range to 0.5, the decline rate of 1 d flexural strength decreased significantly with the increase in GGBS. When the Na_2_O/binder ratio increases from the optimal range to 11% and the SiO_2_/Na_2_O molar ratio from 1.2 to 1.0, the bond strength declines slowly, and high alkalinity is beneficial to the bond strength.
- Considering the coupling effect, the comprehensive optimal of 1 d compressive strength, 1 d flexural strength, and 1 d bond strength was taken as the target. The recommended values of GGBS content, Na_2_O/binder ratio, SiO_2_/Na_2_O molar ratio, and water/binder ratio were 60%, 10.1%, 1.19, and 0.41, respectively. The 1 d compressive strength, 1 d flexural strength, and 1 d bond strength obtained by this ratio were 50.91 MPa, 7.69 MPa, and 5.33 MPa, respectively, and the error was less than 3% compared with the predicted results of the RSM model.
- Comparative tests on MK and GGBS-based geopolymer mortars showed that Mix RSM and Mix GGBS met the requirements of the standard. At 6 h, it had a high compressive strength (18.7 MPa) and bond strength (2.8 MPa) comparable with the GGBS-based geopolymer mortar. Later, it showed even higher late strengths (28 d compressive strength of 88.8 MPa, 28 d bond strength of 7.7 MPa). In particular, Mix RSM had better bond strength than many repair mortars. The shrinkage value of Mix RSM was close to Mix MK, which was 0.05%. Mix RSM had a denser structure, was less water-absorbing, and appeared less efflorescent than Mix MK and Mix GGBS.
- The results of BSE showed that the geopolymer was tightly bound to the matrix. EDS results showed that compared with the geopolymer mortar based on MK and GGBS, Mix RSM had lower Ca/Si at the interface, more C-S-H gel, and less Ca(OH)_2_ at the interface, and the interface was denser.

Research has shown that RSM enables the optimization of the design of geopolymer ratios, but the response of the optimum design is determined by the purpose of rapid repair. In practice, repair mortars must meet a number of requirements, including self-compaction, corrosion resistance, and high abrasion resistance. Proportional optimization with the help of RSM is also required for the performance of geopolymer repair mortars in these application scenarios.

## Figures and Tables

**Table 1:** Chemical compositions of the raw materials (Wt.%).

Raw Materials	SiO_2_	Al_2_O_3_	Fe_2_O_3_	CaO	MgO	K_2_O	SO_3_	Na_2_O	TiO_2_
MK	48.82	41.62	0.53	0.24	2.1	0.19	0.1	0.1	1.21
GGBS	34.29	13.6	0.79	33.16	7.23	0.5	2.17	0.85	0.94

**Table 2:** Size distribution of the precursors.

Cumulative Passing (%)	>100 μm	50–80 μm	20–50 μm	10–20 μm	1.0–10 μm	1.0–5.0 μm	0.5–1.0 μm	0.3–0.5 μm	<0.3 μm
MK	0	0	0	7.57	24.97	46.74	91.28	98.9	100
GGBS	0	0.03	2.55	32.13	60.47	79.45	98.14	100	100

**Table 3:** Physical properties of ISO standard sand.

Fineness Modulus	Density (g/cm^3^)	Cumulative Mass Percentage (%)					
		>2.38 mm	1.18–2.38 mm	0.6–1.18 mm	0.3–0.6 mm	0.15–0.3 mm	<0.15 mm
2.23	2.639	0	19.4	54.8	68.3	79.9	100

**Table 4:** Impact factors and levels in RSM.

Factors	A	B	C	D
1	40%	9%	1	0.4
2	50%	10%	1.2	0.45
3	60%	11%	1.4	0.5

**Table 5:** Geopolymer synthesis mixture design.

Code	A	B	C	D
1	40%	9%	1.2	0.45
2	60%	9%	1.2	0.45
3	40%	11%	1.2	0.45
4	60%	11%	1.2	0.45
5	50%	10%	1	0.4
6	50%	10%	1.4	0.4
7	50%	10%	1	0.5
8	50%	10%	1.4	0.5
9	40%	10%	1.2	0.4
10	60%	10%	1.2	0.4
11	40%	10%	1.2	0.5
12	60%	10%	1.2	0.5
13	50%	9%	1	0.45
14	50%	11%	1	0.45
15	50%	9%	1.4	0.45
16	50%	11%	1.4	0.45
17	40%	10%	1	0.45
18	60%	10%	1	0.45
19	40%	10%	1.4	0.45
20	60%	10%	1.4	0.45
21	50%	9%	1.2	0.4
22	50%	11%	1.2	0.4
23	50%	9%	1.2	0.5
24	50%	11%	1.2	0.5
25	50%	10%	1.2	0.45
26	50%	10%	1.2	0.45
27	50%	10%	1.2	0.45
28	50%	10%	1.2	0.45
29	50%	10%	1.2	0.45

**Table 6:** RSM test results.

Code	$y_{1}/\mathrm{MPa}$	$y_{2}/\mathrm{MPa}$	$y_{3}/\mathrm{MPa}$
1	23.91	4.80	2.36
2	32.45	5.60	4.06
3	31.56	5.30	3.29
4	40.50	7.22	3.85
5	26.85	5.26	4.19
6	35.45	7.30	4.64
7	25.8	5.18	2.55
8	18.48	3.42	1.43
9	32.07	6.82	4.58
10	48.95	7.22	5.58
11	27.55	3.68	1.66
12	33.89	6.63	3.16
13	22.46	4.39	2.67
14	26.20	5.40	3.79
15	17.26	3.67	1.85
16	26.20	4.89	3.08
17	27.78	5.20	2.84
18	40.38	7.24	4.82
19	29.72	5.10	2.48
20	33.52	6.03	3.15
21	25.60	5.23	3.74
22	32.35	6.63	4.94
23	17.16	3.62	1.96
24	22.51	4.64	2.35
25	40.62	7.35	4.47
26	39.99	7.17	4.51
27	37.69	7.44	4.51
28	40.68	7.09	4.27
29	41.87	6.99	4.34

**Table 7:** ANOVA for the regression model and respective model terms.

Source	Df	$1d\;\mathrm{Compressive}\;\mathrm{Strength}\;(y_{1})$				$1d\;\mathrm{Flexural}\;\mathrm{Strength}\;(y_{2})$				$1d\;\mathrm{Bond}\;\mathrm{Strength}\;(y_{3})$			
		MS.	F-Value	*p*-Value	Sig.	MS.	F-Value	*p*-Value	Sig.	MS.	F-Value	*p*-Value	Sig.
Model	14	129.86	65.01	<0.0001	Y	3.35	63.18	<0.0001	Y	2.45	45.19	<0.0001	Y
A	1	271.70	136.02	<0.0001	Y	6.81	128.31	<0.0001	Y	4.58	84.91	<0.0001	Y
B	1	136.55	68.36	<0.0001	Y	3.82	71.96	<0.0001	Y	1.81	33.58	<0.0001	Y
C	1	6.51	3.26	0.0925	Y	0.43	8.02	0.0133	Y	1.49	27.67	0.0002	Y
D	1	260.21	130.26	<0.0001	Y	10.62	200.13	<0.0001	Y	17.67	327.82	<0.0001	Y
A×B	1	0.040	0.020	0.8895		0.31	5.91	0.0291	Y	0.32	6.03	0.0365	Y
A×C	1	19.36	9.69	0.0076	Y	0.31	5.80	0.0303	Y	0.43	7.96	0.0188	Y
A×D	1	27.77	13.90	0.0022	Y	1.63	30.63	<0.0001	Y	0.063	1.16	0.3277	
B×C	1	6.76	3.38	0.0871		0.011	0.21	0.6556		3.025 × 10^−3^	0.056	0.8267	
B×D	1	0.49	0.25	0.6281		0.036	0.68	0.4234		0.16	3.04	0.1227	
C×D	1	63.36	31.72	<0.0001	Y	3.61	68.01	<0.0001	Y	0.62	11.43	0.0066	Y
$A^{2}$	1	7.73	3.87	0.0693		0.22	4.19	0.0598		0.51	9.02	0.0114	Y
$B^{2}$	1	575.99	288.35	<0.0001	Y	12.11	228.21	<0.0001	Y	3.83	69.52	<0.0001	Y
$C^{2}$	1	407.99	204.24	<0.0001	Y	8.66	163.10	<0.0001	Y	4.41	80.20	<0.0001	Y
$D^{2}$	1	222.81	111.54	<0.0001	Y	4.54	85.51	<0.0001	Y	1.11	19.74	0.0008	Y
Residual	14	2.00	-	-	-	0.053	-	-	-	0.061	-	-	-
Lack of Fit	10	1.84	0.77	0.6637	N	0.061	1.77	0.3059	N	0.071	1.96	0.2693	N
Pure Error	4	2.38	-	-	-	0.034	-	-	-	0.036	-	-	-
R^2^		0.9849				0.9844				0.9758			
Adj R^2^		0.9697				0.9688				0.9515			

**Table 8:** Mechanical strength as specified in the standard.

Specification	6 h Compressive Strength	1 d Compressive Strength	28 d Compressive Strength	28 d Flexural Bending Strength
JC/T 2381-2016	≥15 MPa	≥20 MPa	≥30 MPa	≥2.0 MPa

**Table 9:** Bond strengths of different types of repair material.

Samples Used in Previous Studies	Curing Duration	Reported Bond Strength (MPa)
Portland cement (P∙I 42.5) repairing mortar [31]	28 d	5.9
Magnesium phosphate cement mortar [38]	1 d	4.1
Repair mortar containing waterborne epoxy resin emulsions [39]	28 d	7.3
UHPC with 2.5% steel fiber [40]	28 d	5.9
Epoxy resin modified OPC-based mortars [41]	28 d	4.8
FA/BFS repairing mortar (FA:BFS = 3:7) [42]	28 d	6.1
MK-based geopolymer with GGBS replacement of 30% [15]	1 d	3.7
	28 d	6.4
Optimal design of MK-GGBS based geopolymer repairing mortar by RSM (Mix RSM)	6 h	2.8
	1 d	3.8
	28 d	7.7

## Data Availability

All data generated or analyzed during this study are included in this published article.
